# Association of human milk oligosaccharides and nutritional status of young infants among Bangladeshi mother–infant dyads

**DOI:** 10.1038/s41598-022-13296-w

**Published:** 2022-06-08

**Authors:** Sharika Nuzhat, Parag Palit, Mustafa Mahfuz, Md. Ridwan Islam, S. M. Tafsir Hasan, M. Munirul Islam, Shafiqul. A. Sarker, David J. Kyle, Robin L. Flannery, Anita Vinjamuri, Carlito B. Lebrilla, Tahmeed Ahmed

**Affiliations:** 1 Nutrition and Clinical Services Division, International Centre for Diarrhoeal Disease Research, Bangladesh (icddr,b), 68, Shaheed Tajuddin Ahmed Sarani, Mohakhali, Dhaka, 1212 Bangladesh; 2 Office of the Executive Director, International Centre for Diarrhoeal Disease Research, Bangladesh (icddr,b), Dhaka, 1212 Bangladesh; 3 Faculty of Medicine and Life Sciences, University of Tampere, Tampere, Finland; 4 Evolve BioSystems, Inc., Davis, CA 95618 USA; 5 Department of Chemistry, University of California, Davis, CA 95616 USA; 6 Department of Global Health, University of Washington, Seattle, WA USA; 7 James P Grant School of Public Health, BRAC University, Dhaka, Bangladesh

**Keywords:** Diseases, Medical research

## Abstract

Human milk oligosaccharides (HMOs) support the development of a healthy gut microbiome and the growth of infants. We aimed to determine the association of different HMOs with severe acute malnutrition (SAM) among Bangladeshi young infants. This study was nested within a single-blind, randomized, pilot clinical trial (NCT0366657). A total of 45 breastmilk samples from mothers of < 6 months old infants who had SAM (n = 26) or were non-malnourished (n = 19) and were analyzed for constituent HMOs. Of the infants with SAM, 14 (53.85%) had secretor mothers, and 11 (57.89%) of the non-malnourished infants had secretor mothers. A one-unit increase in the relative abundance of sialylated HMOs was associated with higher odds of SAM in age and sex adjusted model (aOR = 2.00, 90% CI 1.30, 3.06), in age, sex, and secretor status adjusted model (aOR = 1.96, 90% CI 1.29, 2.98), and also in age and sex adjusted model among non-secretor mothers (aOR = 2.86, 90% CI 1.07, 7.62). In adjusted models, there was no evidence of a statistically significant association between SAM and fucosylated or undecorated HMOs. Our study demonstrates that a higher relative abundance of sialylated HMOs in mothers’ breastmilk may have a negative impact on young infants’ nutritional status.

## Introduction

Globally 144 million children under the age of five years are stunted, 47 million are wasted and 38 million are overweight^1^. The risk of mortality associated with acute malnutrition is the highest among infants aged under six months^2^. Pre-term birth, sub-optimal breastfeeding, acute infection or congenital anomaly disrupting appetite, and being born small for gestational age or as a twin have been reported as risk factors for infants becoming malnourished^3,4^. The updated WHO guidelines focus on possible initiation and/or re-establishment of exclusive breastfeeding as a management strategy for infant malnutrition^5^. Consequently, an emerging body of evidence suggests that commensal microbial communities, residing primarily in the lower GI tract and collectively known as the “gut microbiome” play an important role in the growth during early life^6^. The GI tract of well-nourished breastfed infants is known to be colonized by lactic acid-producing bacteria (LAB), including *Bifidobacterium* sp. and *Lactobacillus* sp, which confer beneficial effects on the host physiology^7^. Insufficient breast milk intake has been reported among infants suffering from severe acute malnutrition (SAM)^8,9^. Concurrent studies conducted at icddr,b reported chronic depletion of the aforementioned beneficial members of the gut microbiota among children with SAM, which is not replenished upon nutritional rehabilitation following current treatment protocols including low-cost, culturally appropriate, diet for nutritional rehabilitation (halwa and khichuri) for SAM^10^.

Human milk contains a number of bioactive components, among which human milk oligosaccharides (HMOs) have been reported to exhibit pivotal roles in the overall health and nutritional status of infants^11^. Human milk contains human milk oligosaccharide (HMO) more than 20 g/L in colostrum and mature milk contains approximately 12–13 g/L^12^. Also the composition of HMOs vary with maternal nutritional status and dietary intake^13^. The building blocks of these milk oligosaccharides are the five monosaccharides, namely: D-glucose (Glc3), D-galactose (Gal), N-acetylglucosamine (GlcNAc), L-Fucose (Fuc), and sialic acid (Sia; N-acetyl neuraminic acid [Neu5Ac])^11^. Usually, HMOs range from 3 to 8 sugars in size, mostly containing fucose as a constituent although highly complex oligosaccharides containing up to 32 sugars have been reported^14^.

HMOs are not digestible by infants and arrive intact to the large intestine, where they exert prebiotic roles by supporting the development of selected beneficial groups of the gut microbiota, including *Bifidobacterium* sp and some *Lactobacillus* sp. by providing metabolic products for their existence, growth, and ultimate colonization in the gut of infant^15,16^. In addition to helping in healthy gut microbiome, HMOs deliver a number of assistances to infants, including brain development^17^, acting as decoys for harmful organism^18^, and averting disease and infection^19^. Certain HMOs have also been found to reduce frequencies of moderate to severe diarrhea among infants, in particular diarrhea caused by *Campylobacter* and calicivirus^20^.

Human milk oligosaccharides differ between secretor and non-secretor mothers^21^, whereby fucosylation in the human milk oligosaccharides are a result of gene products that regulate Lewis and secretor blood group types^21^. Concurrent shifts in gut microbiota depending on secretor status have been previously reported^22–24^. Subsequently, a Malawian study reported that mothers with undernourished infants had an overall decreased content of milk oligosaccharides^25^. It was also observed that particular human milk oligosaccharides support age-appropriate growth of infants^26^.

A recent study reported that milk oligosaccharides influences the nutritional status of the offspring^27^. However, there has been no report on the variation of HMO content and its effect on the nutritional outcome of young infants, particularly among those of under six months of age. In light of this knowledge gap, we conducted an observational study at the Dhaka Hospital of icddr,b, where we aimed to determine possible relationships between HMO content of breast milk with the nutritional status, anthropometric outcomes, and abundance of *Bifidobacterium infantis* in the gut of these young infants.

## Materials and methods

### Study site and participants

This study was conducted at the Dhaka Hospital of icddr,b. We recruited 49 mothers of infants who were already enrolled in a randomized controlled pilot trial (RCT) entitled “Pilot of a prebiotic and probiotic trial in young infants with severe acute malnutrition (NCT0366657)”. These infants were between 2 and 6 months of age, of either sex, admitted to the Dhaka Hospital of icddr,b due to gastrointestinal or respiratory infection and were categorized into two groups based on their nutritional status: 1) *SAM:* infants with weight for length Z score of less than − 3 (WLZ < − 3) or with the presence of bilateral pedal edema, irrespective of anthropometric measurements and *non-malnourished:* infants with weight for length Z score of greater than − 1 (WLZ > − 1). The final analysis included 45 infants; 4 samples (3 SAM and 1 non-malnourished infant) were excluded because the secretor status of the women could not be determined.

### Collection of breast milk samples

Breast milk samples from mothers were collected under aseptic conditions. Mothers were asked to stop feeding the infant 30 min before sample collection. Iodine-soaked gauze was used to clean the breast. Samples were collected in a sterile bottle using a breast pump. The samples were aliquoted in pre-labeled cryovials and flash-frozen in liquid nitrogen immediately after collection. The samples were transferred to − 80 °C freezers within 24 h of collection and were stored until further analyses.

### HMO extraction and quantitation

The technique of extraction of milk oligosaccharide and quantification has been described in detail in the cited literature^28,29^. In brief, 25 µL aliquots of each breastmilk sample were diluted and defatted via centrifugation. Proteins were precipitated with ethanol and the resulting glycans were reduced with 1M NaBH_4_ for 90 min in a dry incubator set at 65 °C and then purified on solid-phase extraction graphitized carbon cartridges (GCC). The resulting eluents were combined, followed by reconstitution by solvent evaporation and dilution for subsequent analyses. HPLC QqTOF Mass Spectrometry was used for quantitation of the individual HMO constituents, using previously optimized protocols^29^. The specific structures were identified and assigned by matching retention time and exact mass which was matched with the data available in previously developed HMO libraries^30,31^ and the glycans were categorized in the manner as followed in previous publications^28–32^.

### Evaluation of secretor status from breast milk

Secretor status was determined by phenotyping method, as described elsewhere following earlier published methods^33^. Briefly, oligosaccharides with known α(1,2)-Fuc linkages were identified by matching exact mass and retention times with a previously developed in-house library^30,31^. The signal responses of the most abundant α(1,2)-Fuc containing structures, namely 2’-fucosyllactose (2’FL), lactodifucotetraose (LDFT), difucosyllacto-*N*-hexaose a(DFLNHa) and trifucosyllacto-*N-*hexaose (TFLNH), were summed and normalized to the total HMO abundance present in a given sample. Secretor status was assigned based on a previously established and validated threshold^33^. The sum of the relative abundances was obtained and when it exceeded this threshold the mother was deemed to be a secretor; conversely, if it fell below the threshold they were deemed to be non-secretors.

### Identifying human milk oligosaccharides

#### Fucosylated and sialylated HMOs

To identify the HMOs, we followed reports from previously published literature^34^. The compositions of HMOs were given as hexose, N-acetylhexoseamine (HexNAc), fucose, N-acetylneuraminic acid (sialic acid). The compound name of HMOs was expressed with a four-digit number. For example, the compound identifier 4110 indicated that the compound has 4 hexoses, 1 HexNAc, 1 fucose, and 0 sialic acid molecule. Thus, compounds with a nonzero number in the last position are sialylated HMO; those with a nonzero number in the third position are fucosylated HMO.

#### Undecorated HMOs

Other than fucosylated or sialylated HMOs are called undecorated HMOs.

### Evaluation of the B. infantis levels in the fecal samples of the infants at enrolment

Stool samples were collected at baseline, prior to the start of supplementation. Stool samples collected were aliquoted in pre-labeled cryovials and flash-frozen in liquid nitrogen within 20 min after defecation, as practiced in other studies^35^. Aliquots were preserved in − 80 °C freezers until analysis. The laboratory assays were carried out at the Laboratory of Evolve bioSystems, Inc California. The levels of *B.infantis*, the predominant member of the gut flora among young infants was measured from the collected stool samples by qPCR, using protocols described elsewhere^36^.

### Statistical analysis

We presented the characteristics of mothers and infants using percentage and median (interquartile range (IQR)), as appropriate. To assess the association of the relative abundance of different HMOs with severe acute malnutrition, simple and multivariable binary logistic regression models were used separately for fucosylated, sialylated, and undecorated HMOs. As the sample size is relatively small, we used firth logistic regression instead of conventional logistic regression. Firth logistic regression provides more reliable estimates in instances of small sample size, complete separation, and rare events using penalized maximum likelihood estimation^37^. We expressed the strength of association as odds ratio (OR) and adjusted odds ratio (aOR) with a 90% confidence interval (90% CI) for each unit increase in the relative abundance of a certain HMO.

Since this is an exploratory study, to better understand the relationship between the relative abundance of the HMOs and SAM, we fitted infant age- and infant sex-adjusted models, and infant age-, infant sex-, and mother’s secretor status-adjusted models. We also fitted infant age- and infant sex-adjusted models separately for secretor and non-secretor mothers. To examine the discriminative performance of the logit models for the detection of SAM, the area under the receiver operating characteristic (AUROC) curves was estimated^38^.

All statistical tests were two-sided. Since the study is pilot in nature, the statistical significance was evaluated at *p* < 0.10, and 90% CIs was reported^39^. Data analysis was done in Stata v15.1 (Stata Corp, College Station, TX, USA). Z scores for nutritional status were calculated using WHO Anthro software.

### Ethical approval

The randomized controlled trial was registered at ClinicalTrials.gov (NCT03666572) on 12/09/2018 (https://clinicaltrials.gov/ct2/show/NCT03666572) and the research protocol was approved by the Institutional Review Board (IRB) of International Centre for Diarrhoeal Disease Research, Bangladesh (icddr,b) on 16 February, 2018 (approval number: PR- 17112). All clinical aspects of the study were supervised by the investigators and study physicians. All activities were conducted in accordance with the Helsinki Declaration of 1975, revised in 1983. Names of the participants were de-identified prior to analysis.

### Patient consent for publication

Details that disclose the identity of the participants or their information were kept confidential. All procedures were approved by the appropriate ethics committee and have therefore been performed in accordance with the ethical standards laid down in the 1964 Declaration of Helsinki and its later amendments. Informed consent was taken prior to sample collection and intervention.

## Results

We analyzed a total of 45 breast milk samples, of which 26 (26/45, 57.7%) were from the mothers of SAM infants and 19 were from mothers of non-malnourished infants (19/45, 42.2%). Among the mothers of SAM infants, 14 were secretors (14/26, 53.85%) and 12 were non-secretors (12/26,46.15%). Among the mothers of non-malnourished infants, 11 were secretors (11/19, 57.89%) and 8 were non-secretors (8/19, 42.11%).

In comparison to the mothers of non-malnourished infants, the mothers of the SAM infants were younger in age (23.42 years vs. 25.89 years), had lower educational qualification (38.46% vs. 21.05%, education level less than 5 years), had babies delivered with lower birth weight (2.76 kg vs. 3.04 kg) and lower proportion of term infant (65.38% vs. 89.74%). Higher proportion of cesarean section (LUCS) was predominant among the SAM infants in comparison to the non-malnourished infants (50% vs. 21.05%). Moreover, SAM infants presented with a short duration of diarrheal illness, when compared to the non-malnourished infants (4.46 days vs. 7.26 days). Antibiotic use before hospitalization was significantly higher in non-malnourished infants (34.78% vs. 94.44%, p < 0.01). Fecal *B. infantis* levels baseline were lower in non-malnourished infants compared to the SAM infants (6.00 vs. 4.88 Log10 CFU/µg of DNA), but this difference was not statistically significant (Table 1).

**Table 1:** The baseline characteristics of SAM and non-malnourished young infants and their mothers.

Characteristics	SAM (n = 26)	Non-malnourished (n = 19)	*P* value
Maternal age in years (mean, SD)	23.42 ± 5.32	25.89 ± 5.43	0.135
Maternal education (less than 5 years) (n, %)	10 (38.46%)	4 (21.05%)	0.213
Gestational age (in weeks) (mean, SD)	37.35 ± 2.24	38.53 ± 1.93	0.071
Term infant (gestational age ≥ 37 weeks) (n, %)	17 (65.38%)	17 (89.47%)	0.063
Mode of delivery- by C-section (n, %)	13 (50%)	4 (21.05%)	0.048
Birth weight in kg (mean, SD)	2.76 ± 0.68/17	3.04 ± 0.54/14	0.229
Male infant (n, %)	16 (61.54%)	13 (68.42%)	0.634
Age on admission (in days) (mean, SD)	103.58 ± 35.09	131.74 ± 29.30	0.007
Edematous infant (n, %)	17 (65.38%)	NA	NA
WAZ of infant on admission (mean, SD)	-3.41 ± 0.63/9	− 0.70 ± 1.03/19	< 0.001
WLZ of infant on admission (mean, SD)	− 3.80 ± 0.33)/9	0.23 ± 0.79/19	< 0.001
LAZ of infant on admission (mean, SD)	− 1.31 ± 1.25	− 1.17 ± 1.08	0.699
Diarrheal duration of infant on admission (in days) (mean, SD)	4.46 ± 7.01	7.26 ± 6.68	0.184
Volume of breast milk intake in percentage/day feed (mean, SD)	19.85 ± 14.43	97.74 ± 9.86	< 0.001
Use of antibiotic prior to admission (n, %)	8 (34.78%)	17 (94.44%)	< 0.001
Admission *B.infantis* level in stool sample (mean, SD) (Log10 CFU/µg of DNA)	6.00 ± 2.27/23	4.88 ± 3.56/19	0.225
Secretor mothers (n, %)	14 (53.85%)	11 (57.89%)	0.78
Sialylated HMOs (% of relative abundance) (mean, SD)	6.18 ± 2.21	3.88 ± 0.99	< 0.001
Fucosylated HMOs (% of relative abundance) (mean, SD)	54.38 ± 9.41	59.45 ± 7.04	0.055
Undecorated HMOs (% of relative abundance) (mean, SD)	39.44 ± 8.34	36.67 ± 6.93	0.244

HMOs, human milk oligosaccharides; WAZ, weight for age z score; WLZ, weight for length z score; LAZ, length for age z score.

**Figure 1:**
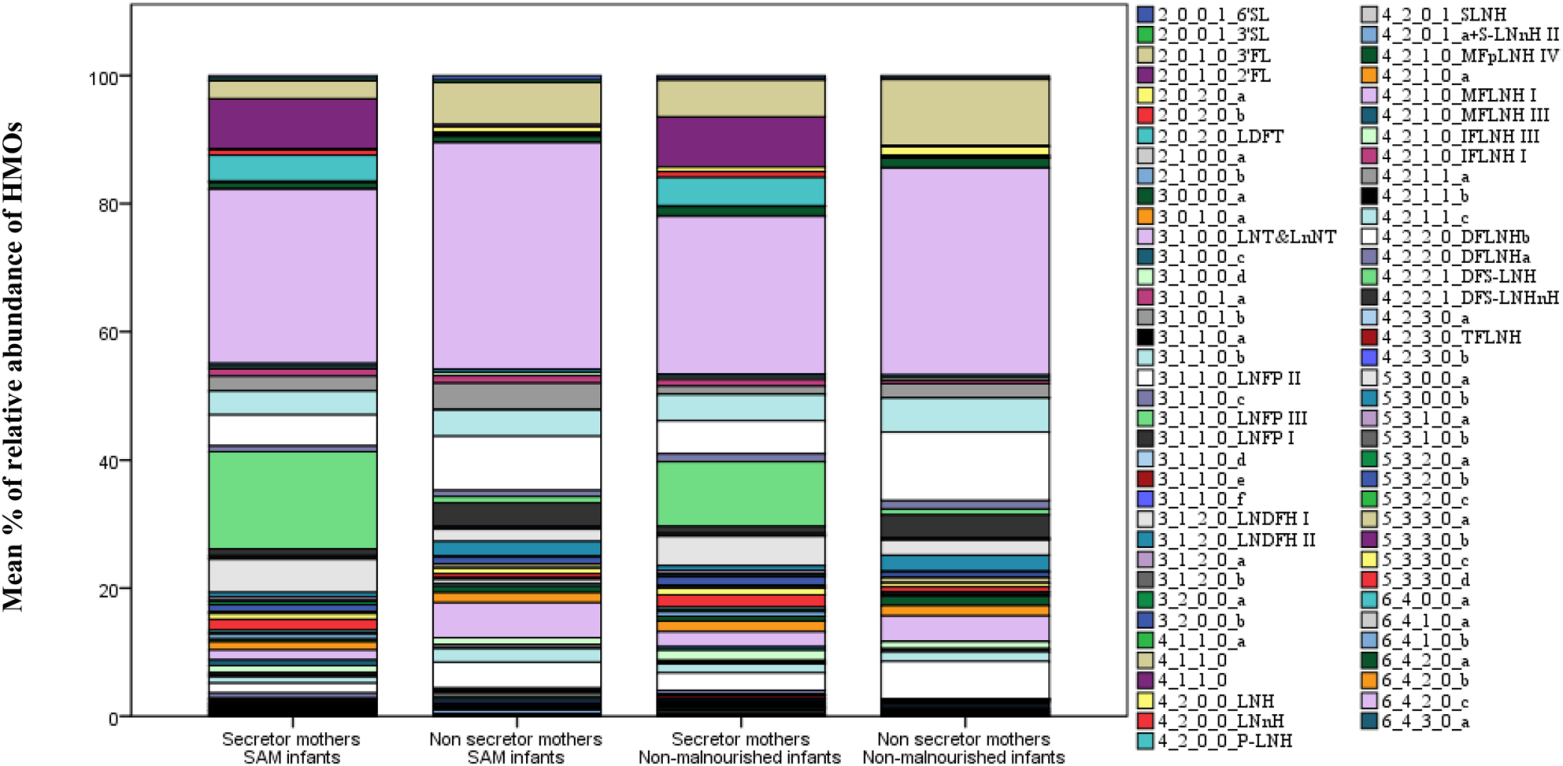
Distribution of different HMOs in mothers of different secretor status and having infants with different nutritional status. [6′SL, 6′-Sialyllactose; 3′SL, 3′-Sialyllactose; 3′FL, 3′-Fucosyllactose; 2′FL, 2′-Fucosyllactose; LDFT, lactodifucosyllactose; LNT&LnNT, lacto-N-tetraose& lacto-N-neotetraose; LNFP II, Lacto-N-fucosylpentose-II; LNFP III, Lacto-N-fucosylpentose-III; LNFP I, Lacto-N-fucosylpentose-I; LNDFH I,lacto-N-difucosylhexose-I; LNDFH II, lacto-N-difucosylhexose-II; LNH, lacto-N-hexaose; LNnH, lacto-N-neohexaose; MFLNH I, monofucosyllacto-N-hexaose I; MFLNH III, monofucosyllacto-N-hexaose III; IFLNH III, isomer 3 fucosyl-paralacto-Nhexaose; IFLNH I, isomer 1 fucosyl-paralacto-N-hexaose; P-LNH, para-lacto-N-hexaose; SLNH, Monosialyllacto-N-hexaose; a+S-LNnH II, No literature name + sialyllacto-N-neohexaose II; MFpLNH IV, Monofucosyl-paralacto-N-hexaose; DFLNHb, Difucosyllacto-Nhexaose b; DFLNHa, Difucosyllacto-Nhexaose a; DFS-LNH, Difucosylmonosialyllacto-*N*-neohexaose; DFS-LNHnH, Difucosylmonosialyllacto-N-neohexaose; TFLNH, Trifucosyllacto-N-hexaose.]

Figure 1 shows the distribution of individual oligosaccharides in breastmilk of mothers of SAM and non-malnourished infants. In the analysis, we identified 73 HMOs out of which 26 HMOs had a chemical name and others only had a compound identifier.

In Table 2 shows the association of malnutrition status of the young infants with different types of HMOs. Sialylated HMOs were associated with higher odds of severe acute malnutrition status in age and sex adjusted model (AOR = 2.00, 90% CI 1.30, 3.06), in age, sex, secretor status adjusted model (AOR = 1.96, 90% CI 1.29, 2.98) and also among non-secretor mothers when age and sex adjusted model was used (AOR = 2.86, 90% CI 1.07, 7.62). All these different statistical models show statistically significant association with sialylated HMO and severe acute malnutrition among the young infants. Fucosylated HMOs were less likely associated with severe acute malnutrition but there was no significant association between these. Undecorated HMOs showed association with higher odds severe acute malnutrition among the young infants in all the models but associations were not statistically significant. In Supplementary Table, we included the conventional logistic regression.

**Table 2:** Association of sialylated, fucosylated, and undecorated HMOs with severe acute malnutrition among young infants using firth logistic regression.

	Sialylated HMOs			Fucosylated HMOs			Undecorated HMOs		
	aOR	90% CI	*P* value	aOR	90% CI	*P* value	aOR	90% CI	*P* value
Model 1	2.00	1.30–3.06	0.008	0.95	0.89–1.02	0.224	1.03	0.96–1.10	0.486
Model 2	1.96	1.29–2.98	0.008	0.95	0.88–1.02	0.242	1.03	0.95–1.10	0.570
Model 3	1.52	0.90–2.57	0.192	0.98	0.88–1.08	0.710	1.00	0.90–1.12	0.944
Model 4	2.86	1.07–7.62	0.078	0.94	0.85–1.04	0.285	1.03	0.94–1.14	0.557

For model 1: adjusted odds ratio (aOR) (90% CI) was adjusted for age and sex.

For model 2: adjusted odds ratio (aOR) (90% CI) was adjusted for age and sex and secretor status.

For model 3: it was for secretor mothers only and adjusted odds ratio (aOR) (90% CI) was adjusted for age and sex.

For model 4: it was for non-secretor mothers only and adjusted odds ratio (aOR) (90% CI) was adjusted for age and sex.

Abbreviations: aOR, adjusted odds ratio; CI, confidence interval, HMO, human milk oligosaccharide.

Different adjusted models showed modest to strong ability to distinguish association with different HMOS and severe acute malnutrition (AUROC range: 0.74–0.86) (Supplementary Figs. 1,2,3).

## Discussion

The study was conducted among 45 infant-mother dyads. The data presented here were based on the HMO analysis of the breast milk of mothers who had either non-malnourished or SAM infants. In the study, we observed that the association of sialylated HMOs with severe acute malnutrition among young infants. Conversely, fucosylated HMOs and undecorated HMOs had no significant association with severely malnourished infants. To our knowledge, ours is the first study to assess the relation of different HMOs with malnutrition status among Bangladeshi infant-mother dyads without any supplementation of HMOs.

Breast milk contains complex oligosaccharides that escape digestion by the intestinal enzymes and transit through the gastrointestinal tract of the infant^40^. The variety of HMOs appears to be endless; to date over 200 different structures of HMOs have been identified in breast milk. HMO profile is affected by host secretor status, encoded by fucosyltransferase 2 (*FUT2*) gene, based on the secretor/Lewis blood group^21,41^. Secretor status phenotyping depends upon the expression of the histo blood group antigens (HBGAs): fucosyltransferase FUT2 (secretor gene), and FUT3 (Lewis gene) in secretions such as saliva and breast milk^42^. These antigens may act as innate host factors that differentially influence the susceptibility of individuals and their offspring^43^. The analytical procedure that was used in this study for the secretor status phenotyping of the mothers involves the measurement of a certain level of HBGAs in the breast milk samples, using a set cut-off value^33^. FUT2 and FUT3 genes that are rendered non-functional due to distinct polymorphisms are thus unable to express the corresponding HBGAs in the secretions^44^. Henceforth, the secretor status of such individuals with non-functional FUT2 and FUT3 genes cannot be determined by analytical methods, as used in our study. This is a probable explanation of their “Undetermined” secretor status.

The oligosaccharide abundance in breast milk is also affected by maternal genetics, particularly by the FUT2 gene, which encodes an enzyme accountable for the addition of a fucose residue at the α1-2 position on a backbone of abundant glycans containing galactose^45^. The absence or trace amount of α1-2 fucosylated oligosaccharides is responsible for the decreased amount of HMOs in non-secretor mothers’ breast milk^33^.

In our study, we have observed an association of higher odds of severe acute malnutrition among the young infants with sialylated HMOs and this association was statistically significant. On the contrary, fucosylated HMOs were less likely to be associated with severely malnourished infants. Recent studies demonstrated that human milk oligosaccharides (HMOs) are linked to growth in early infancy. In a study conducted among 37 mother-infant dyads, it was observed that an increase in lacto-N-fucopentaose (LNFP) I was linked with lower infant weight at 1 and at 6 months and with lower lean and fat mass at 6 months. Further, At 6 months, LNFP II and disialyl-lacto-N-tetraose (DSLNT) were related to higher fat mass^46^. The amount of fucosylation in breast milk changes over the course of lactation^47^, which may affect the protection deliberated to an infant. HMOs containing α1,2-fucosyl linkages have been revealed to help the growth of members of the *Bifidobacterium* genus due to prebiotic action^48^.

Different HMOs have been shown to exert an impact on the weight-for-age z score (WAZ) of infants, particularly 3’-SL, as observed in the Gambian study^49^. In this study, the relative sialylation of HMOs was not a predictor for WAZ of the infants^49^. However, in our study, we observed that severely malnourished infants consumed breast milk which had significantly higher sialylated HMOs than non-malnourished infants. Sialylated HMOs was also found to have impact on linear growth in animal model study^50^. The report from a Malawian cohort showed that among only the secretor mothers, the total abundance of HMOs was positively associated with increments in length-for-age z-score (LAZ) from 6 to 12 months of age^51^. Analysis of HMOs of two Malawian birth cohort also reported that severely stunted infants had significantly low sialylated HMO^25^. Observation of this study was also used in animal model study which indicated relationship between sialylated HMO and growth promotion^25^. On enrolment our study infants were with severe acute malnutrition, so we could not follow them to get association of linear growth with sialylated HMOS. In our model, there was significant association with sialylated HMOs with severe acute malnutrition both in age, sex-adjusted model and age, sex, secretor status adjusted model. Comparatively lower sample size in this study may have concealed the significant effect of fucosylated HMOs on the nutritional status of the infants enrolled in our study. Recent review article reported that the results of different studies on HMO were also contradictory due to mixed interpretations^52^. With our exploratory study we have found association of sialylated HMOs with severe acute malnutrition but we could not find causal association from this cross-sectional design.

The infant's intestinal microbiome predominantly consists of two subspecies of *Bifidobacterium longum*: subsp. *infantis* (*B. infantis*) and subsp. *longum* (*B. longum*). Competitive growth of *B. infantis* in the neonatal intestine has been connected to the use of human milk oligosaccharides (HMO)^53^. Previous studies have shown that different fucosylated HMOs have an impact on the incidence of diarrhea^49^. Our study infants exhibited diarrhea on admission, so we were unable to establish a specific role of any of the constituent HMOs on diarrheal incidences among these young infants with different nutritional statuses. We have observed that malnourished infants presented with shorter duration of diarrhea compared to non-malnourished infants. Malnourished infants present with increased disease severity due to their impaired immune system which could have an impact on early presentation of such infants^54^.

Fucosylated HMOs and N-glycans on milk proteins are beneficial for the growth of healthy gut microbiota, acting as prebiotics^25,48^. Changes in gut microbiota depending on secretor status have been reported earlier^22–24^. The impact of maternal secretor status in microbiota is well documented and affects different bacterial groups during lactation^55^. Different studies reported that HMOs in breast milk are key factors which promote the development of members of the *Bifidobacterium* genus in the gut^56,57^. *Bifidobacterium longum* subsp. *infantis* (*B. infantis*) is unique among other gut microbes as this members of this particular subspecies of *B.longum* areequipped with the appropriate machinery to consume the full range of human milk oligosaccharides (HMOs)^58^. A large proportion of breast milk comprises fucosylated HMOs which can also be utilized by select *B. breve* strains and *B. bifidum* through extracellular fucosidase activity^59,60^. The gut microbiota is often exposed to a variety of antimicrobial agents, like antibiotics, which are used to combat bacterial infection in humans^61^. This antibiotic therapy may cause depletion of the beneficial members of the gut microbiota, a phenomenon known as “dysbiosis’^62^. Results from our study suggest that different types of HMOs consumed by infants have an impact on the nutritional status of the infants. However, the use of antibiotic may have masked the effect of HMOs on gut microbiota especially of the *Bifidobacterium* species which showed that even with exclusive breastfeeding, the levels of *Bifidobacterium infantis* was comparatively lower in non-malnourished infants. This association explains context-specific correlation, which also has causational support. Different studies also reported that infants without exposure to antibiotics presented with a higher percentage of *Bifidobacteriaceae*^63,64^. A number of metagenomics studies have illustrated associations between the altered gut microbiota and child malnutrition. Early depletion of *Bifidobacterium longum* appears to be the first step in gut microbiota alteration in severe acute malnutrition^65,66^ since any change in the relative abundance of *Bifidobacterium* in early infancy marks a change in the infant gut microbiome, potentially indicating dysbiosis.

The smaller sample size of this study makes it underpowered for certain study findings. Nevertheless, differences were detected in the relative abundance of particular HMO constituents in the breast milk of mothers of infants of different nutritional statuses, and as a result, the concentration of each constituent HMO was not measured. Moreover, the SAM infants were only partially breastfed and this variable might have had influenced the overall study findings and we could not assess the total amount of HMOs. A prospective large data set is required to have clear understanding on relationship of HMOs with ponderal as well as linear growth of infants.

This study provides primary observational data that the nutritional status of infants is affected by breast milk HMO composition of mothers and that additional supplementation of certain deficient HMO along with *B. longum* subsp *infantis* could be an intervention approach for the management of malnutrition in early infancy.

## Supplementary Information

Supplementary Information 1.

Supplementary Information 2.

Supplementary Information 3.

Supplementary Information 4.

## Data Availability

This data set contains some personal information of the study patients (such as name, admission date, month, area of residence). Our IRB has required that the personal information of the participants is not disclosed. Thus, the policy of our centre (icddr,b) is that we should not make the availability of whole data set in the manuscript, the supplemental files, or a public repository. However, data related to this manuscript are available upon request and for researchers who meet the criteria for access to confidential data may contact with Armana Ahmed (armana@icddrb.org) to the Research Administration of icddr,b (http://www.icddrb.org/).
