# Supplementary material for: Association of human milk oligosaccharides and nutritional status of young infants among Bangladeshi mother–infant dyads
Source: Sci Rep. 2022 Jun 8;12:9456. doi: 10.1038/s41598-022-13296-w (PMC9177541; doi:10.1038/s41598-022-13296-w)
Supplement: Supplementary file 1 — Supplementary Information 1. [file 41598_2022_13296_MOESM1_ESM.docx]

Supplementary table 1: Association of sialylated, fucocylated and undecorated HMOs with severe acute malnutrition using logistic regression

|  | Sialylated HMOs | | | Fucosylated HMOs | | | Undecorated HMOs | | |
| --- | --- | --- | --- | --- | --- | --- | --- | --- | --- |
|  | AOR | 95% CI | P value | AOR | 95% CI | P value | AOR | 95% CI | P value |
| Model 1 | 2.24 | 1.28-3.93 | 0.005 | 0.94 | 0.87-1.03 | 0.186 | 1.03 | 0.95-1.13 | 0.449 |
| Model 2 | 2.25 | 1.28-3.93 | 0.005 | 0.94 | 0.85-1.03 | 0.201 | 1.03 | 0.93-1.14 | 0.535 |
| Model 3 | 1.67 | 0.82-3.40 | 0.155 | 0.97 | 0.84-1.12 | 0.703 | 1.00 | 0.87-1.16 | 0.952 |
| Model 4 | 21.32 | 0.21-2146.60 | 0.194 | 0.91 | 0.79-1.05 | 0.214 | 1.05 | 0.91-1.21 | 0.494 |

For model 1: adjusted odds ratio (aOR) (95% CI) was adjusted for age and sex

For model 2: adjusted odds ratio (aOR) (95% CI) was adjusted for age and sex and secretor status

For model 3: it was for secretor mothers only and adjusted odds ratio (aOR) (95% CI) was adjusted for age and sex

For model 4: it was for non-secretor mothers only and adjusted odds ratio (aOR) (95% CI) was adjusted for age and sex

Abbreviations: aOR, adjusted odds ratio; CI, confidence interval; HMO, human milk oligosaccharide
