# Supplementary material for: Association of human milk oligosaccharides and nutritional status of young infants among Bangladeshi mother–infant dyads
Source: Sci Rep. 2022 Jun 8;12:9456. doi: 10.1038/s41598-022-13296-w (PMC9177541; doi:10.1038/s41598-022-13296-w)
Supplement: Supplementary file 2 — Supplementary Information 2. [file 41598_2022_13296_MOESM2_ESM.pdf]

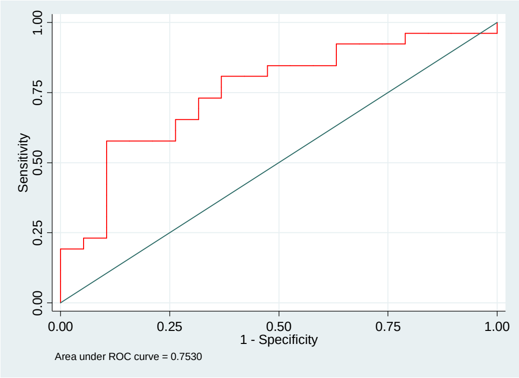


Fig 1 (a)

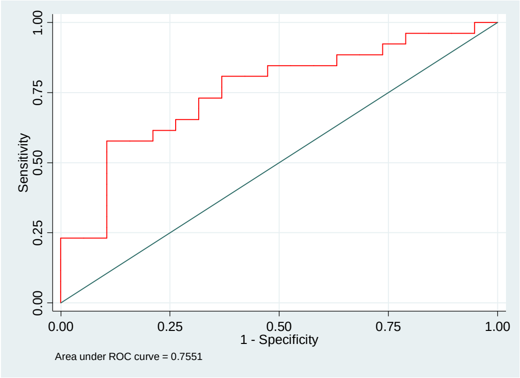


Fig 1 (b)

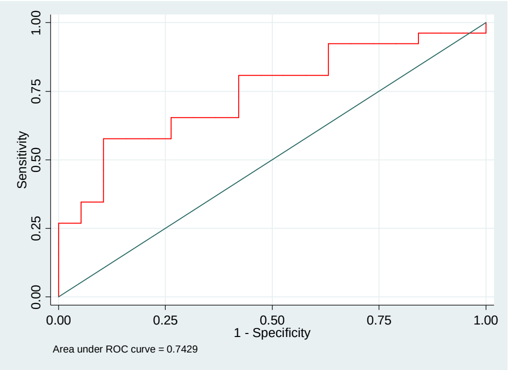


Fig 1 (c)

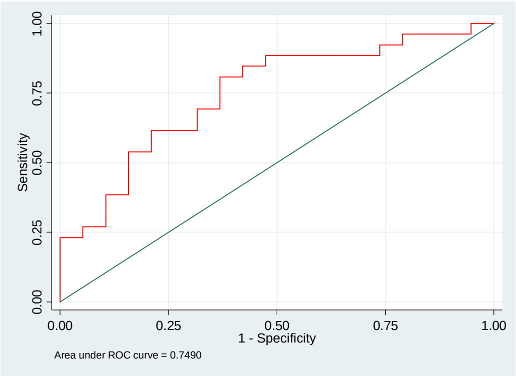


Fig 1 (d)

Fig 1: Receiver operating characteristic curves of different logit models for detecting association of fucosylated HMOs with severe acute malnutrition, (a) for model 1: adjusted odds ratio (aOR) (90% CI) was adjusted for age and sex, (b) for model 2: adjusted odds ratio (aOR) (90% CI) was adjusted for age and sex and secretor status, (c) for model 3: it was for secretor mothers only and adjusted odds ratio (aOR) (90% CI) was adjusted for age and sex, (d) for model 4: it was for non-secretor mothers only and adjusted odds ratio (aOR) (90% CI) was adjusted for age and sex. Abbreviations: ROC, receiver operating characteristic; HMO, human milk oligosaccharide
