# Supplementary material for: Association of human milk oligosaccharides and nutritional status of young infants among Bangladeshi mother–infant dyads
Source: Sci Rep. 2022 Jun 8;12:9456. doi: 10.1038/s41598-022-13296-w (PMC9177541; doi:10.1038/s41598-022-13296-w)
Supplement: Supplementary file 4 — Supplementary Information 4. [file 41598_2022_13296_MOESM4_ESM.pdf]

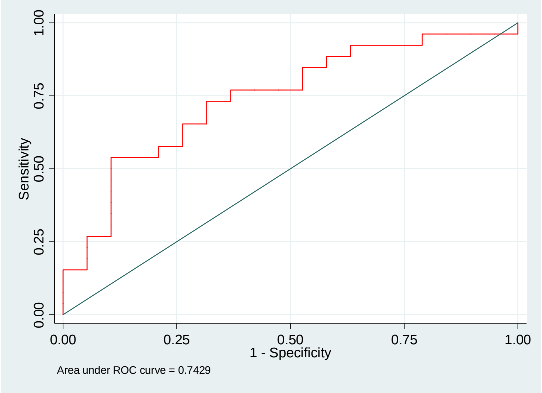

Fig 3 (a)

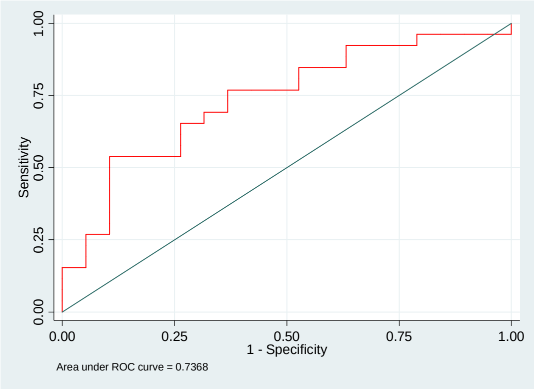

Fig 3 (b)

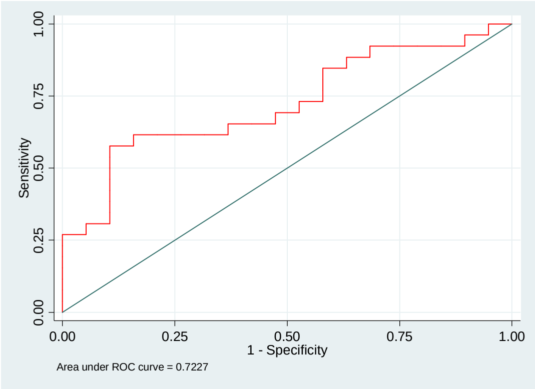

Fig 3 (c)

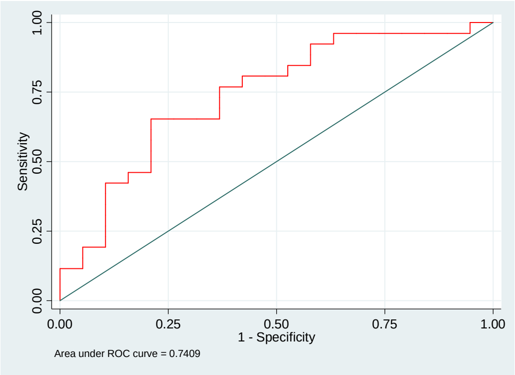

Fig 3 (d)

Fig 3: Receiver operating characteristic curves of different logit models for detecting association of undecorated HMOs with severe acute malnutrition, (a) for model 1: adjusted odds ratio (aOR) (90% CI) was adjusted for age and sex, (b) for model 2: adjusted odds ratio (aOR) (90% CI) was adjusted for age and sex and secretor status, (c) for model 3: it was for secretor mothers only and adjusted odds ratio (aOR) (90% CI) was adjusted for age and sex, (d) for model 4: it was for non-secretor mothers only and adjusted odds ratio (aOR) (90% CI) was adjusted for age and sex. Abbreviations: ROC, receiver operating characteristic; HMO, human milk oligosaccharide
